# Travel Health Alert Notices and Haiti Cholera Outbreak, Florida, USA, 2011

**DOI:** 10.3201/eid1711.110721

**Published:** 2011-11

**Authors:** Monica U. Selent, Amanda McWhorter, Valery M. Beau De Rochars, Rebecca Myers, David W. Hunter, Clive M. Brown, Nicole J. Cohen, Noelle A. Molinari, Kirsten Warwar, Danisha Robbins, Katherine E. Heiman, Anna E. Newton, Ann Schmitz, Michael J. Oraze, Nina Marano

**Affiliations:** Centers for Disease Control and Prevention, Atlanta, Georgia, USA (M.U. Selent, A. McWhorter, V.M. Beau De Rochars, R. Myers, D.W. Hunter, C.M. Brown, N.J. Cohen, N.A. Molinari, K. Warwar, D. Robbins, A. Schmitz, N. Marano); Atlanta Research and Education Foundation, Decatur, Georgia, USA (K.E. Heiman, A.E. Newton); US Customs and Border Protection, Washington, DC, USA (M.J. Oraze)

**Keywords:** Haiti, cholera, travel, health communication, disease outbreaks, information dissemination, bacteria, health education, travel medicine, Florida

## Abstract

To enhance the timeliness of medical evaluation for cholera-like illness during the 2011 cholera outbreak in Hispaniola, printed Travel Health Alert Notices (T-HANs) were distributed to travelers from Haiti to the United States. Evaluation of the T-HANs’ influence on travelers’ health care–seeking behavior suggested T-HANs might positively influence health care–seeking behavior.

Travel health alert notices (T-HANs) have been used since the 1970s by the Centers for Disease Control and Prevention (CDC; Atlanta, GA, USA) as a communication tool for international travelers arriving in the United States during public health emergencies. T-HANs, typically printed on yellow cards, inform travelers about possible disease exposures, advise them to seek health care if symptoms develop, and instruct them to give the T-HAN to their physicians. T-HANs also provide clinical guidance and resources. Despite their repeated use, to our knowledge the influence of T-HANs on travelers’ health care–seeking behavior during an outbreak has not been evaluated.

After a large cholera outbreak was confirmed in Haiti on October 21, 2010, CDC immediately began providing health recommendations to travelers and guidance to US clinicians, primarily through the CDC website and other electronic means (*1**,**2*). By early December, 5 imported cholera cases with *Vibrio cholerae* isolates identical to the Haiti strain were confirmed in Florida; 2 case-patients had been discharged from emergency departments without cholera diagnoses, requiring subsequent reevaluation and hospitalization (*3*).

The rapidly escalating epidemic in Haiti and the historically high travel volume between Haiti and the United States during December and January prompted CDC to distribute T-HANs to travelers from Haiti to reduce the risk for delayed health care. Typically, 8–10 direct flights from Haiti arrive daily in the United States at 4 airports: Miami and Fort Lauderdale, Florida; New York (John F. Kennedy International), New York; and San Juan, Puerto Rico. Miami receives approximately half of these flights (*4*). The cholera T-HAN was written in plain language; translated to French, Haitian-Creole, and Spanish (Figure); and distributed by US Customs and Border Protection (CBP) officers at passport control booths at these 4 airports. T-HANs were not distributed to passengers on connecting flights from Haiti because these flights had fewer passengers from Haiti and flew to numerous US cities, making T-HAN distribution impractical.

**Figure:**
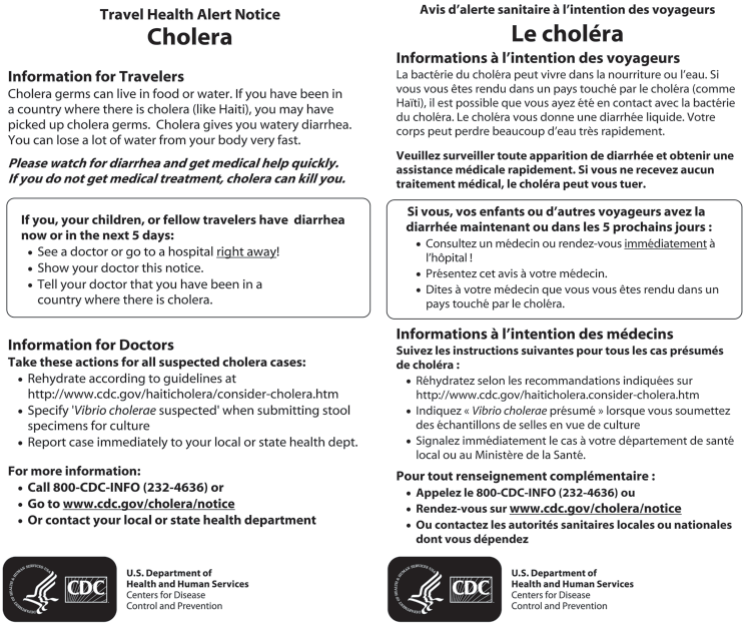
Travel health alert notice for January 2011 Haiti cholera outbreak showing English and French versions. Haitian-Creole and Spanish versions were printed on the reverse side (not shown).

## The Evaluation

We evaluated the effectiveness of T-HANs through 3 methods. First, we counted the number of page views at a unique Internet address (printed only on the T-HAN and unlikely to be indexed in search engines, which redirected to CDC’s Haiti Cholera Web page). Second, on January 10–11, 2011, a voluntary 5-question survey was administered to travelers from Haiti at the Miami airport. Travelers were asked whether they had received and read the T-HAN, their need for cholera health information, their likelihood of seeking health care if they had onset of diarrhea within 5 days after arrival, and whether their travel had originated in the United States. The survey was administered orally in English or Haitian-Creole by trained interviewers in the airport’s Federal Inspection Station ≈10–20 minutes after T-HAN distribution. Analyses were adjusted for respondents’ sex and travel origin. Third, US cholera case-patients from Haiti who traveled to the 4 airports during the T-HAN distribution period were asked by their respective health departments whether they had received a T-HAN and whether it had influenced their decision to seek health care.

From December 20, 2010, through March 31, 2011, ≈73,500 T-HANs were distributed at the 4 airports, 51,500 (70%) in Miami. Seventy-five redirects were counted at the T-HAN Web address, half within the first month. T-HAN distribution was not associated with increased calls to CDC’s information hotline (printed on the T-HAN) or traffic on the CDC website.

Of 1,348 travelers from Haiti who arrived in Miami on January 10–11, 2011, a total of 882 (65%) were surveyed (Table). Receiving or reading the T-HAN was significantly associated with reported need for cholera health information (adjusted prevalence ratio 1.27 and 1.16, respectively; p<0.05). T-HAN readers were more likely than nonreaders to indicate that they were likely to seek health care for diarrhea (adjusted prevalence ratio 1.05; p = 0.0127). Of 7 confirmed cholera case-patients who met criteria for inclusion, 2 received T-HANs; both indicated that the T-HAN influenced their decision to seek health care.

**Table:** T-HAN survey of 1,348 travelers from Haiti at the Miami International Airport, Florida, USA, January 10–11, 2011*

Characteristic	No. (%)
Total travelers surveyed	882 (65)
Male travelers surveyed	504 (57)
Survey responses	
Received T-HAN	664 (75)
Read T-HAN	245 (28)
Reported need for cholera information	458 (52)
Trip originated from United States	675 (77)
Likelihood of seeking care if diarrhea developed in next 5 d	
Likely	693 (79)
Uncertain	97 (11)
Not likely	89 (10)

*T-HAN, travel health alert notice.

## Conclusions

This evaluation suggests that T-HANs had a small positive influence on travelers’ health care–seeking behavior. Although more than half of survey respondents reported a need for cholera information, and receiving or reading the T-HAN was associated with this need, the low number of redirects on the T-HAN website suggests that most recipients did not use the T-HAN as a source for more information, possibly because the T-HAN information was sufficient or because they sought information elsewhere. In developing a T-HAN, complex scientific information must be reframed into simple, concise messages that grab travelers’ attention. Translation for non–English-speaking travelers often is required, and imagery for lower literacy audiences might be needed. T-HANs also must raise clinicians’ suspicion for uncommon communicable diseases; guide testing, treatment, and reporting in accordance with public health recommendations; and remain valid as these recommendations evolve.

Rapid T-HAN distribution to travelers during a public health emergency poses unique logistic, legal, and political challenges. CBP’s assistance in distributing cholera T-HANs was invaluable; however, achieving widespread distribution was difficult. CBP officers have multiple responsibilities when reviewing travelers’ documents. CBP’s opinion was that T-HAN distribution was more successful during the pandemic (H1N1) 2009 outbreak than during the Haiti cholera response because T-HANs were distributed to all travelers rather than to a specific subset. With the many international travelers processed each day, an automated prompt on their computers could assist CBP officers to consistently distribute T-HANs to specific travelers.

In-flight T-HAN distribution has been explored as an alternative to postarrival distribution. However, numerous difficulties complicate CDC's ability to secure agreements with airlines, including positioning T-HANs on aircraft with changing flight plans and airlines’ concerns about negative public perceptions and possible legal and economic ramifications. Another option is predeparture distribution (e.g., in Haiti), but CDC lacks authority to require distribution of health information to US-bound travelers overseas. Public announcements on airplanes and electronic messaging or posters in airports require advance planning with airlines and airport officials and should be pursued as possible alternatives. To encourage appropriate health care visits and medical assessments, future evaluations also should assess the effectiveness of pretravel and posttravel health messages on social media sites, the CDC Travelers’ Health website (www.cdc.gov/travel), and messaging aimed at clinicians.

Our results are subject to several limitations. The relatively low response rate, which reflects the operational difficulties of conducting surveys in airports, limits traveler representativeness. Interviewer or cultural bias also might have been present. Although the survey was voluntary, it was conducted in the airport Federal Inspection Station and therefore might reflect approval-seeking bias. Additionally, estimation of the T-HANs’ effectiveness could have been biased because few travelers read the T-HAN, possibly because of lack of time, intent, or ability to read it (because the T-HAN was not tested with lower literacy audiences). The small number of cholera case-patients who received T-HANs might not have accurately reflected the T-HAN’s effect on health care–seeking behavior, and no information was available for travelers with diarrhea who did not seek health care or in whom cholera was not diagnosed.

The Haiti cholera T-HAN response was relatively small; during a larger scale event, resource requirements for T-HAN distribution would be far greater. Given the logistical challenges of T-HAN distribution, further efforts are warranted to study the effectiveness of T-HANs and to identify alternative methods of providing health information to travelers.
